# Comparison of postoperative effectiveness of less invasive short external rotator sparing approach versus standard posterior approach for total hip arthroplasty

**DOI:** 10.1186/s13018-020-02188-2

**Published:** 2021-01-11

**Authors:** Tianbao Wang, Yongwei Zhou, Xiaofei Li, Siqi Gao, Qining Yang

**Affiliations:** 1 The Orthopedics Department, Affiliated Jinhua Hospital, Zhejiang University School of Medicine, No. 365 Renmin East Road, Jinhua City, 321000 Zhejiang Province China; 2 The Orthopedics Department, Wuyi TCM Hospital Medical Community, No.186 Wuyang East Road, Jinhua City, 321000 Zhejiang Province China

**Keywords:** Total hip arthroplasty, Short external rotator sparing, Standard approach, Prospective study

## Abstract

**Background:**

Most of the studies assessing the corrective posterior total hip arthroplasty (THA) mainly focused on the mini-incision approach. Studies exploring the short external rotator sparing approach are rare. Therefore, this study aimed to compare the effectiveness of standard posterior approach and short external rotator sparing approach.

**Methods:**

This prospective observational study included 126 patients who underwent THA in June 2017–June 2018. Patients were assigned to standard (standard posterior approach) and corrective (short external rotator sparing approach) groups based on the surgical method. Surgical data were recorded postoperatively. Postoperative hip joint recovery was assessed using the times to ambulation and independent stair use, and Western Ontario and McMaster Universities Osteoarthritis Index (WOMAC) score, Harris score, and Oxford hip score (OHS) at 2 and 8 postoperative weeks. The visual analog scale (VAS) was used for postoperative pain assessment.

**Results:**

Postoperative changes of creatine kinase (CK), myoglobin, CRP, and prosthesis position were similar in both groups (*P* > 0.05). However, intraoperative blood loss (*P* < 0.001) and postoperative 6-h drainage volume (*P* = 0.03), hospital stay, blood transfusion rate, and times to ambulation and independent stair use were significantly reduced in the corrective group. Postoperatively, Oxford, and WOMAC scores significantly decreased in both groups. After surgery, the VAS score was more overtly decreased in the corrective group compared with the standard group.

**Conclusions:**

This study concluded that the less invasive short external rotator sparing approach for THA caused less damage, reducing perioperative blood loss, shortening functional recovery time, maintaining prosthesis stability, and improving postoperative pain.

## Background

Various kinds of approaches to the hip joint have been proposed for total hip arthroplasty (THA), including the posterior (Moore or Southern), lateral (Hardinge), anterolateral (Watson Jones), direct anterior approach (Smith-Peterson), and posterolateral approaches [1, 2]. Of these, the posterolateral approach has been widely applied and has several advantages, such as sufficient exposure of the acetabular fossa and femur, and the preservation of abductor muscles in THA [3, 4]. On the other hand, recently, corrective surgical approaches for THA have attracted increasing attention from surgeons due to the advantages of mini-incision and rapid postoperative recovery [5, 6]. These modifications could be broadly classified as mini-incision and external rotator sparing approaches [5–7].

However, no universally accepted standards are currently available for the exploration of corrective approaches. In addition, studies assessing corrective posterior THA mostly focused on the mini-incision approach, with only a few exploring the short external rotator sparing approach. Interestingly, a matched cohort study performed in 2010 showed that the corrective approach sparing the quadrate muscle of thigh could reduce intraoperative blood loss, alleviate postoperative resting pain, and shorten postoperative rehabilitation time; however, indicators of intraoperative inflammation, such as C-reactive protein (CRP) and creatine kinase (CK), did not change significantly. The corrective surgical approach sparing the piriformis muscle resulted in longer distance in the 6-min walking test and higher degree of patient satisfaction compared with the standard approach; whereas, the acetabular anteversion angle was not significantly different between the two groups. In addition, the patients who underwent corrective surgical approach had advantages of reduced intraoperative injuries and faster postoperative recovery [8]. However, these studies indicated that the surgery was highly challenging and suggested the surgery was not worth applying, especially in obese patients, for short-term benefits [9–11]. Additional reports suggested that the surgical apparatuses and dilators used in the corrective surgeries induced soft tissue and muscle contusions. Hence, the corrective surgical approach could only provide limited additional benefits, with no objective clinical evidence supporting the advantages [12–14].

The above findings clearly indicate that the benefits of corrective surgeries in THA remain unknown. Therefore, the present prospective cohort study aimed to compare the effectiveness of standard posterior approach with short external rotator sparing approach.

## Methods

### Patients

This prospective study included consecutive patients who underwent THA in the Orthopedics Department of Jinhua Central Hospital between June 2017 and June 2018. Inclusion criteria were 1) age ≥ 18 years; 2) THA for diseases, such as osteonecrosis of the femoral head, hip osteoarthritis, and femoral neck fracture; and (3) no previous hip arthroplasty. Exclusion criteria were 1) traumatic arthritis; 2) language or communication difficulties, or psychiatric disorders that could not be followed-up; 3) concurrent severe internal diseases or severe osteoporosis; 4) BMI > 30; and 5) old acetabulum fractures accompanied by pelvic deformities or acetabular defects. The patients were assigned to the corrective and standard groups based on the applied surgical methods. The patients in this study were on the same degeneration stage (IV stage) according to Kellgren–Lawrence (K–L) grading scale [15]. This study was approved by the Ethics Committee of Jinhua Central Hospital (approval number LGF19H060005). All of the patients included in this study provided signed informed consent.

### Surgical methods

All surgeries were performed by the same surgical team comprising a chief surgeon (15 years of experience) and two attending surgeons (6 years of experience). All patients were implanted a cementless prosthesis (Zimmer, USA) under general anesthesia through tracheal intubation. The blood lost during the and within 6 h postoperatively was collected using an autologous blood recovery system, and the volume of blood loss was recorded. In patients with intraoperative blood loss > 500 mL, autologous blood transfusion was conducted. The patient grouping was done according to the administered surgical approach, which was based on the disease condition of the patient. However, the standard surgery was suggested for obese patients.

### Surgery in the standard group

In the standard group, the patient was first administered general anesthesia and then placed in the lateral position on the unaffected side, followed by routine disinfection and draping. A line of about 14 cm was drawn from the proximal end of the greater trochanter of the femur to the distal end. Next, the skin, subcutaneous tissues, and fascia were incised layer by layer, and blunt dissection of the gluteus maximus was performed. Next, the hip joint was slightly internally rotated to expose the piriformis muscle, the internal obturator muscle, the gemellus superior and inferior, and the quadrate muscle of the thigh. Muscle terminations were resected with an electric scalpel, and the muscles were folded upward to expose the articular capsule. Then, a T-shaped incision was made to cut open the articular capsule. The hip joint was then dislocated and the femoral neck was resected at 1 cm above the lesser trochanter. Next, the femoral head was retrieved with a special apparatus, with the femoral neck trimmed to an appropriate length. After clearing the acetabular margin, the ligamentum capitis femoris was resected and residual soft tissues in the occipital area were cleared to expose the osseous acetabulum. Acetabular prostheses of different sizes were implanted to determine the ideal match and the osseous coverage. An appropriate prosthesis was selected and placed in the acetabular cup at the position of 45° abduction and 15° anteversion, and screws were used for fixation if necessary. The affected limb was upheld and kept adducted as much as possible. Grooving and reaming were performed at the proximal end of the femur to obtain the ideal size, and the testing model was placed. The femoral head was implanted, and hip joint reduction was performed. The lower leg length, range of motion, and hip joint stability were examined.

### Surgery in the corrective group

In the corrective group, after general anesthesia, the patient was placed in the lateral position on the unaffected side, and routine disinfection and draping were performed. A posterolateral incision was made on the affected hip. Then, an oblique, arch-shaped incision of about 14 cm was made posterior to the greater trochanter of the femur. Next, the skin, subcutaneous tissues, and the fascia were incised, and the quadrate muscle of the thigh, the inferior gemellus, and the distal internal obturator muscle were resected along the posterior margin of femoral tuberosity. The internal obturator muscle was vertically incised, and an L-shaped incision was made for the articular capsule. The tendon of the piriformis muscle, the gemellus superior, the upper part of the internal obturator muscle, and the posterosuperior articular capsule were preserved. Later, the procedure described for the standard group was performed, with hip joint dislocation by internal rotation, hip bending, and knee bending. Then, osteotomy was performed at the femoral neck, while the femoral calcar was preserved, and the femoral head was retrieved with a special apparatus. The cavitas glenoidalis and the round ligament were resected, and the acetabular margin was cleared to expose the osseous acetabulum. Acetabular prostheses were implanted to determine the ideal match, and screws were used for fixation, if necessary. The affected limb was upheld and kept adducted as much as possible with grooving and reaming at the proximal end of the femur to obtain the ideal size. Then, the testing model was placed. The femoral head was implanted, and hip joint reduction was performed. The lower leg length, range of motion, and hip joint stability were examined.

### Data collection and follow-up

The baseline data of patients in both groups, including sex, age, body mass index (BMI), initial diagnosis, American Society of Anesthesiologists Classification (ASA) score (assessed according to the patient’s condition and surgical risk before anesthesia) [16], visual analog scale (VAS) score for pain, and Western Ontario and McMaster Universities Osteoarthritis Index (WOMAC) score, and Oxford hip score (OHS), were collected. Intra- and postoperative parameters in both groups, including incision length, operation time, intraoperative blood loss volume, blood transfusion volume, postoperative drainage volume, and hospital stay, were recorded. The degree of injury before and at 48 h postoperatively, as well as changes in inflammation-related indicators, including creatine kinase (CK), myoglobin, and 72-h postoperative C-reactive protein (CRP), versus preoperative levels were also recorded. Postoperative parameters, including times to bedside ambulation, independent stair use, and joint dislocation rates were recorded at 8 weeks postoperatively. Pain intensity (1 to 7 days postoperatively) was assessed using the VAS. The WOMAC score was used to assess the severity of arthritis and effects of the treatment preoperatively and at 8 weeks postoperatively, according to symptoms and signs of the patient [17]. The Harris score and OHS score were used to assess the recovery of hip joint functions. X-ray and CT were performed to assess the position of the prosthesis after operation [18].

The patients were followed-up twice through clinical visits or telephone calls at 2 and 8 weeks postoperatively. The Harris score, OHS, WOMAC score, X-ray film, and CT scan were assessed and recorded during each follow-up session.

### Statistical analysis

SPSS22.0 (IBM, Armonk, NY, USA) was used for statistical analysis. GraphPad Prism 7.0 (GraphPad, San Diego, USA) was used for graphing. Continuous variables with normal distribution were represented using mean ± standard deviation (SD) and those with skewed distribution were described as median and range. Independent samples *t* test was performed for comparisons between the two groups. The chi-squared test was carried out for comparing categorical data. Multi-factor analysis of variance was adopted for assessing the postoperative Harris, WOMAC, and Oxford scores, as well as VAS score on day 7 postoperatively. *P* < 0.05 was considered statistically significant.

## Results

### Baseline patient data

This study included 126 patients with a median follow-up time of 8 weeks (2–14 weeks). Of these, 54 patients, including 28 male (51.8%) and 26 female (48.2%) underwent standard THA, and were aged 68 ± 6 years. Forty-six (85.2%) of them had osteonecrosis of the femoral head, four (7.4) had hip osteoarthritis, and four (7.4) were femoral neck fracture cases. The Crown stage of the patient with congenital hip dysplasia was < grade I; the lengths of bilateral limbs were identical with no gluteus atrophy, and the disease condition was mild. WOMAC score (*P* = 0.03), Oxford score (*P* = 0.04), CK (*P* = 0.04), and myoglobin (*P* = 0.04) were significantly different between the two groups (Table 1).

**Table 1:** Baseline and preoperative characteristics of the patients

	Corrective group (*n* = 72)	Standard group (*n* = 54)	*P*
Age (year), mean ± SD	65.6 ± 5.8	66.4 ± 7.7	0.76
Male, *n* (%)	34 (47.2)	28 (51.8)	0.02
BMI	24.7 (3.9)	22.1 (4.7)	0.04
Initial diagnosis, *n* (%)			0.49
Osteonecrosis of the femoral head	57 (79.2)	46 (85.2)	
Hip osteoarthritis	6 (8.3)	4 (7.4)	
Femoral neck fracture	8 (11.1)	4 (7.4)	
Congenital hip dysplasia	1 (1.4)	0	
ASA score, *n* (%)			0.53
1	6 (8.3)	4 (7.4)	
2	41 (56.9)	30 (55.6)	
3	25 (34.7)	20 (37.0)	
CK, mean ± SD	126 ± 36.9	109 ± 29.4	0.04
Myoglobin, mean ± SD	48.0 ± 17.7	36.0 ± 14.7	0.04
CRP, mean ± SD	4.8.0 ± 1.9	4.6 ± 2.5	0.39

*ASA score* American Society of Anesthesiologists Classification score, *BMI* body mass index, *CK* creatine kinase, *CRP* C-reactive protein

### Surgery-related data

The lengths of the incisions made during the surgery were not significantly different between the corrective and standard groups. However, operation time (*P* < 0.001), intraoperative blood loss (*P* < 0.001), and postoperative 6-h drainage volume (*P* = 0.03) were less in the corrective group. In addition, the number of patients who required autologous blood transfusion was less in the corrective group compared with those of the standard group (*P* = 0.03). The intraoperative data for both standard and corrective groups are given in Table 2.

**Table 2:** Intraoperative and postoperative patient data

	Corrective group (*n* = 72)	Standard group (*n* = 54)	*P*
**Intraoperative**			
Incision length (cm), mean ± SD	13.8 ± 0.9	13.4 ± 0.7	0.4
Operation time (min), mean ± SD	51 ± 11.7	45 ± 10.3	< 0.001
Intraoperative blood loss (mL), mean ± SD	278.4 ± 132.7	349.6 ± 189.1	< 0.001
Postoperative 6-h drainage (mL), mean ± SD	290.7 ± 174.3	509.6 ± 280.7	0.03
Number of patients requiring autologous blood transfusion, *n* (%)	5 (6.9)	13 (24.1)	0.03
Location of prosthesis (°), mean ± SD			
Anteversion angle	42.6 ± 6.7	43.1 ± 6.2	0.53
Abduction angle	20.7 ± 4.8	19.9 ± 4.2	0.48
**Postoperative**			
CK, mean ± SD			
1st day postoperative	559.1 ± 361.7	578.9 ± 476.1	0.71
2nd day postoperative	436.3 ± 265.8	395.4 ± 293.7	0.21
Myoglobin, mean ± SD			
Change at 1 day postoperatively	178.9 ± 147.3	199.5 ± 11.6	0.43
Change at 2 days postoperatively	62.9 ± 54.2	76.3 ± 33.5	0.26
CRP change at 3 days postoperatively, mean ± SD	79.1 ± 47.5	82.5 ± 39.6	0.63
Time to ambulation (days), mean ± SD	3.0 ± 2.3	4.1 ± 2.2	0.04
Distance in 6-min walking test (m), mean ± SD	29 ± 9.7	27 ± 7.9	0.68
Time to using stairs independently (days), mean ± SD	5.5 ± 2.0	7.2 ± 2.7	0.02
Hospital stay (days), mean ± SD	9.2 ± 3.1	10.8 ± 3.8	0.62
Joint dislocation, *n* (%)	1 (1.4)	2 (3.7)	0.399

*CK* Creatine kinase, *CRP* C-reactive protein

CK and myoglobin at 1st and 2nd day postoperatively were not significantly different between the two groups (*P* > 0.05). Idem for CRP at 3 days postoperatively (*P* > 0.05).

### Postoperative recovery of the hip joint

After surgery, the distances in the 6-min walking test were not significantly different between the two groups. However, times to bedside ambulation (*P* = 0.04) and independent stair use (*P* = 0.02) were significantly short in the corrective group compared with the standard group. Moreover, no statistically significant difference was observed in the joint dislocation rates in the standard and corrective groups. The postoperative data for both standard and corrective groups are given in Table 2.

### Comparison of hip joint functions and quality of life

**Fig. 1:**
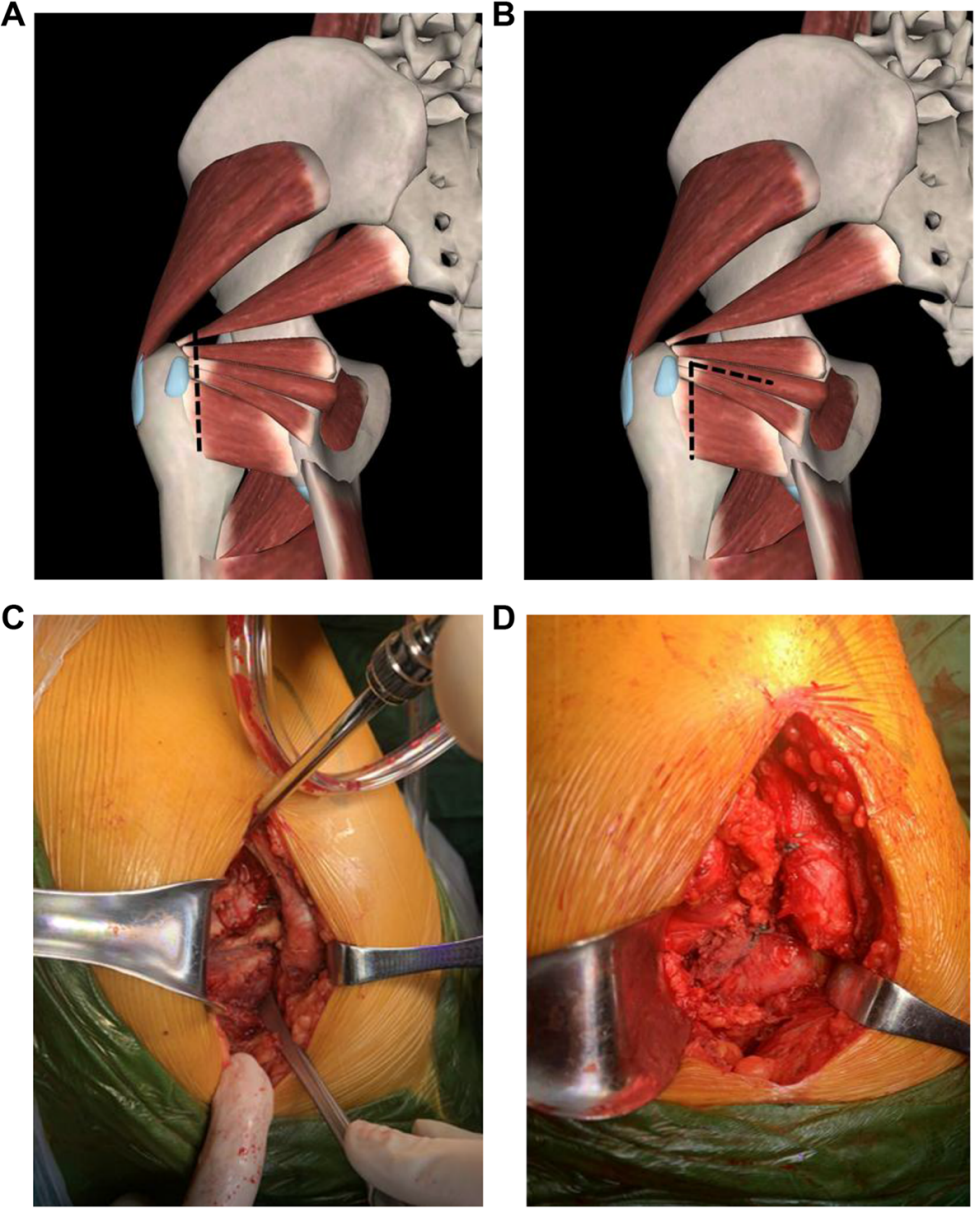
Surgical approaches in surgical methods. Virtual images of **a** standard surgery: cutting off the piriformis, obturator internus, gemellus superior and inferior, and quadratus femoris. Virtual images of **b** corrective surgery: transection of the quadratus femoris, inferior gemellus, and distal obturator internus; longitudinal splitting of the obturator internus; and conserving the piriformis tendon, superior gemellus, and upper part of the obturator internus. Real images of **c** external rotator sparing approach in corrective surgery. Real images of **d** after repairing in corrective surgery

Compared with the preoperative values, Harris scores (*P* = 0.007) and Oxford scores (*P* = 0.047) were significantly decreased in both groups after surgery. At 2 weeks postoperatively, Harris scores (*P* = 0.003) and Oxford scores (*P* < 0.001) improved in the corrective group. However, both groups showed similar values at 8 weeks postoperatively. Multivariate analysis of variance showed that there were statistically significant differences in the effects of surgical methods on hip function score (Harris score, *P* = 0.007; Oxford score, *P* = 0.047) (Table 3, Fig. 1a, b).

**Table 3:** Comparison of hip joint functions between two groups

	Corrective group (*n* = 72)	Standard group (*n* = 54)	*P*
Harris score			
Preoperative	40.1 ± 7.7	39.5 ± 8.2	0.986
2 weeks postoperatively	80.2 ± 10.3	73.7 ± 12.3	0.003
8 weeks postoperatively	91.3 ± 12.7	89.2 ± 13.5	0.636
*P*	0.007		
OHS score			
Preoperative	43.7 ± 7.4	40.7 ± 13.4	0.043
2 weeks postoperatively	30.4 ± 9.3	37.2 ± 10.2	< 0.001
8 weeks postoperatively	25.3 ± 8.2	27.5 ± 9.7	0.562
*P*	0.047		
WOMAC score			
Preoperative	49.7 ± 10.7	44.3 ± 14.9	0.131
2 weeks postoperatively	30.7 ± 12.2	37.6 ± 16.2	0.031
8 weeks postoperatively	21.3 ± 16.9	23.9 ± 18.7	0.706
*P*	0.379		
VAS score			
Preoperative	3.32 ± 1.73	3.26 ± 1.42	0.999
24 h postoperatively	2.21 ± 1.35	2.9 ± 1.21	0.001
48 h postoperatively	1.62 ± 0.87	1.95 ± 0.73	0.352
3 days postoperatively	0.92 ± 0.61	1.42 ± 0.64	0.043
7 days postoperatively	0.45 ± 0.48	0.96 ± 0.59	0.037
*P*	< 0.001		

*OHS score* Oxford hip score, *VAS* visual analog scale, *WOMAC score* Western Ontario and McMaster Universities Osteoarthritis Index score

WOMAC scores in both groups were decreased significantly after surgery; they were also significantly lower in the corrective group at 2 weeks postoperatively (Table 3).

Based on VAS scores, postoperative hip joint pain was significantly reduced. In addition, pain at 24 h, 3 days, and 7 days after surgery (*P* = 0.001, 0.043, and 0.037, respectively) were significantly reduced in the corrective group compared with that of the standard group (Table 3). Multivariate analysis of variance showed that the corrective surgical approach had substantial effects on postoperative pain improvement (*P* < 0.001).

## Discussion

The current study demonstrated that THA via the short external rotator sparing approach causes less damage and decreases perioperative blood loss compared with the standard approach, shortening functional recovery time, maintaining prosthesis stability, and improving pain postoperatively.

The corrective posterolateral approach in THA has several advantages, including relatively easy operation, clearly exposed surgical field, minimal injuries to soft tissues, and improved preservation of abductor muscles [19], whereas the standard posterolateral approach damages the continuity between the posterior external rotators and the greater trochanter of the femur, resulting in a higher postoperative dislocation rate compared with the anterior approach [20, 21]. However, in this study, patients were restricted to perform activities for 3 months after surgery and the joint anteversion angle was increased during surgery [22], which increased the stability of the hip joint. Hence, the dislocation rates were not significantly different between the standard and corrective groups.

Previous studies assessing the posterolateral approach in THA mostly investigated the differences between the mini-incision and standard surgeries [23, 24]. In addition, operations with external rotator sparing mainly preserved the quadrate muscle of the thigh [8, 25, 26] or the piriformis muscle [9, 11], while those sparing a larger extent of the short external rotator are scarce. In contrast to mini-invasive surgeries pursuing small incisions, muscle-sparing operations do not necessarily minimize the incisions, resulting in more indications. For instance, such surgeries could be performed in obese patients, and incisions could be expanded according to intraoperative conditions.

The articular capsule and inter- and extra-articular ligaments of the hip, as well as the surrounding muscles play important roles in stabilizing the hip joint [27]. In general, external rotators, including the piriformis muscle, the internal obturator muscle, and the gemellus superior, are resected in standard THA. Despite muscle suturing and reconstruction later during surgery, the trauma and inflammatory damage remain more pronounced in the standard approach than that observed with the external rotator sparing approach. A randomized, controlled clinical trial of piriform-sparing THA showed that corrective surgery not only has the advantages of conventional posterolateral approach surgeries, but also involves the concept of mini-invasiveness. Moreover, no osteotomy of the greater trochanter or resection of the piriformis muscle was required during the operation, and only piriformis muscle terminations on the articular capsule were dissected, resulting in significantly less trauma, reduced postoperative blood loss, and shortened hospital stay [11]. In our study, the corrective surgery largely preserved the short external rotators, including the tendon of the piriformis muscle, the upper part of the internal obturator muscle, and the gemellus superior. Moreover, intra- and postoperative blood loss was reduced, fewer patients required autologous blood transfusion, and hospital stay was shortened significantly, confirming the advantages of corrective surgeries sparing the short external rotators to a large extent.

In this study, the time to hip joint recovery was shorter in the corrective group compared to that of the standard group. In addition, bedside ambulation and independent stair use occurred earlier in the corrective group. Damage to the short external rotators and postoperative pain was reduced in the corrective group compared with that of the standard group. These findings were in agreement with that of the piriformis-sparing studies by Khan et al. [11]. The tendon of the piriform muscle was resected in the standard surgery, with suturing adding to the tension of the ischiadic nerve; meanwhile, postoperative inflammatory edema of the muscles further increased the muscular tension, which finally increased the duration of postoperative pain. The hip joint function and patient condition scores were decreased in both groups after operation compared with the presurgical values. However, these scores showed no significant difference between the corrective and standard groups postoperatively. These findings corroborated the results of the study by Khan et al., which also showed that hip joint function and patient condition scores in the corrective group were different from those of the standard group at 6 weeks postoperatively but without statistical significance [28].

Incision sizes in both groups were similar; in addition, CK and myoglobin changes on the first and second days after surgery were not significantly different between the two groups. CKP and myoglobin have been used as indicators of muscle damage severity, while CRP amounts reflect the severity of inflammation [29, 30]. However, the actual values of such indicators in surgical trauma remain unclear. Multiple studies have shown that surgical trauma differs in patients administered distinct modifications of THA, while increase in CKP and myoglobin are not significantly different [31] corroborating the current findings. These changes could be induced by contusion injuries due to the apparatuses (e.g., retractors and dilators) used perioperatively in both groups.

This study has few limitations. First, this study is of a prospective observational nature with an evidence level inferior to that of a randomized controlled clinical trial. However, consecutive patients treated in our hospital between 2017 and 2018 were included, and the current findings could reflect the actual clinical situation. However, since it was also a single-center study with a relatively small sample size, the results were hard to be generalized. Moreover, as it was a non-randomized study, the patient demographics are different between the two groups, even though the difference at baseline was consistent with the reality of the study. Hence, a randomized trial will be required in future for further investigation of this aspect. Another limitation was the shorter follow-up period. Therefore, large multi-center randomized clinical trials with long-term follow-up are required to confirm our results.

## Conclusions

In summary, corrective THA sparing short external rotators could reduce the damage to the nerves and soft tissues, decrease the perioperative blood loss, shorten the functional recovery time, maintain the prosthesis stability, alleviate the hip joint pain, and accelerate the postoperative hip joint recovery, thereby improving short-term patient condition.

## Data Availability

The datasets used and/or analyzed during the current study are available from the corresponding author on reasonable request.

## Abbreviations

ASA: American Society of Anesthesiologists Classification
BMI: Body mass index
CK: Creatine kinase
CRP: C-reactive protein
OHS: Oxford hip score
THA: Total hip arthroplasty
VAS: Visual analog scale
WOMAC: Western Ontario and McMaster Universities Osteoarthritis Index
